# Genetic Mapping and QTL Analysis of Fruit Traits in Melon (*Cucumis melo* L.)

**DOI:** 10.3390/cimb45040224

**Published:** 2023-04-14

**Authors:** Haiyong Zhao, Taifeng Zhang, Xiaobing Meng, Jiayan Song, Chen Zhang, Peng Gao

**Affiliations:** 1 College of Horticulture and Landscape Architecture, Northeast Agricultural University, No. 600, Changjiang Road, Harbin 150030, China; 2 Key Laboratory of Biology and Genetic Improvement of Horticulture Crops (Northeast Region), Ministry of Agriculture and Rural Affairs, Harbin 150030, China

**Keywords:** QTL mapping, linkage map, exocarp firmness, edible pericarp firmness, soluble solid content

## Abstract

Melon (*Cucumis melo* L.) is an important horticultural cash crop and its quality traits directly affect consumer choice and market price. These traits are controlled by genetic as well as environmental factors. In this study, a quantitative trait locus (QTL) mapping strategy was used to identify the potential genetic loci controlling quality traits of melons (i.e., exocarp and pericarp firmness and soluble solid content) based on newly derived whole-genome single nucleotide polymorphism-based cleaved amplified polymorphic sequence (SNP-CAPS) markers. Specifically, SNPs of two melon varieties, M4-5 and M1-15, as revealed by whole-genome sequencing, were converted to the CAPS markers, which were used to construct a genetic linkage map comprising 12 chromosomes with a total length of 1414.88 cM, in the F_2_ population of M4-5 and M1-15. The six identified QTLs included: *SSC6.1* and *SSC11.1* related to soluble solid content; *EF12.1* associated with exocarp firmness; and *EPF3.1*, *EPF3.2* and *EPF7.1* related to edible pericarp firmness. These genes were located on five chromosomes (3, 6, 7, 11, and 12) in the flanking regions of the CAPS markers. Moreover, the newly developed CAPS markers will be useful in guiding genetic engineering and molecular breeding in melon.

## 1. Introduction

Melon (*Cucumis melo* L.) is a Cucurbitaceae vegetable crop widely cultivated in many countries, including China. In fact, melons constitute an important component of fruit and vegetable production in China [1]. Melon quality is determined by various characteristics [2]; for instance, melon is popular for its sweet taste, pleasant flavor, and high nutritional value [3]. The sensory quality of melon fruit depends largely on its soluble solid content (SSC) and its volatile aromatic components. In addition, melon fruit has a high SSC, including organic acids, multiple vitamins, and soluble proteins [4,5,6], which not only determines melon flavor but also is commonly used for the quality evaluation of melon. One of the most crucial characteristics related to SSC is the sugar concentration [6] as sugar is the main component that affects the quality and flavor of melon fruit, and is also the basic raw material for the synthesis of vitamins, pigments and aromatic substances. In addition, sugar provides the osmotic catalyst for fruit cell enlargement [7].

The firmness of melon fruit refers to the resistance of the flesh to external pressure. It is essential for sensory attributes, and can suit different needs of consumers, and thus has been used as one of the indicators of melon quality [8]. As a typical quantitative trait affected by genetic and environmental factors, the firmness trait of melon is quite complex. It can be affected by germplasm genetic differences and is typically manifested as the fruit harvest and ripening index [9,10]. The firmness of melon can be affected by cellular contents, such as intercellular space, cellulose, and starch contents. During the melon fruit ripening and softening process, the respiration rate increases, the starch and other substances are degraded, and pectin, which plays an essential role in the cell wall support, is decomposed into fructose, glucose, and other polysaccharide substances, resulting in reduced fruit firmness [11].

These traits have shown great variation since domestication, and their genetic bases have been extensively studied [12,13]. Multiple molecular tools have been developed to help researchers map traits in many crop varieties and link them to certain genomic loci or genes [14,15], thus accelerating plant breeding. The use of molecular markers has facilitated the unraveling of molecular mechanisms underlying different traits [16,17]. The identified markers have accelerated genetic crop breeding based on marker assisted selection (MAS). Several genetic maps of melon have been established since Pitrat [18] first used morphological markers to create a linkage map of melon [19,20,21]. QTL analysis has been used to link genomic regions to fruit traits, such as form, dimension, firmness, weight, and SSC. For instance, Argyris et al. identified 78 QTLs related to the traits of sugar and organic acid contents using near-isogenic and hybridized melon lines [6]. Paris et al. [22] identified 57 QTLs in 81 melon recombinant inbred lines (RILs) that are related to multiple quality traits, including 10 related to SSCs. In another study, 27 QTLs were linked to traits including shape, size, and pulp content in the F_2_ population of melon [23].

The next–generation sequencing (NGS) method has been widely used to understand the genetic mechanisms regulating melon fruit diversity [24,25]. The Spanish Institute of Agricultural Sciences successfully constructed the first melon reference genome (the double-haploid line DHL92 of *C. melo* ssp. *Melo*) in 2012 [26]. In 2020, the latest version of the melon reference genome (Melon (DHL92) v4) was released [27]. This improved melon genome assembly has greatly facilitated the designing of high-density genetic markers [28].

In this study, the F_2_ populations of the thick-skinned melon (M4-5, low SSC) and thin-skinned melon (M1-15, high SSC) were used to construct the CAPS markers, which were further used to identify QTLs related to melon quality traits. Based on previous studies that deployed CAPS markers to elucidate melon fruit quality traits [29], we further assessed the effectiveness of SNP-CAPS markers and using this method, we successfully identified a QTL (*SSC6.1*) that is associated with the trait of soluble solid content in melon. Thus, this study provides further knowledge on marker-assisted breeding of specific fruit varieties.

## 2. Materials and Methods

### 2.1. Plant Materials

P_1_ (M4-5) has a round shape, low SSC, and slow growth rate; P_2_ (M1-15) has an oval shape, high SSC, and fast plant growth rate. Usage of the P_1_ (M4-5, female) and P_2_ (M1-15, male) seeds was approved by the Key Laboratory of Biology and Genetic Improvement of Horticultural Crops (Northeast Region), Ministry of Agriculture, Northeast Agricultural University, China. P_1_ (M4-5, female) and P_2_ (M1-15, male) were crossed to generate the F_1_ generation. F_1_ was further selfed to obtain the F_2_ generation. In a greenhouse at Northeast Agricultural University’s Xiangyang Experiment Agricultural Station in Harbin, China (44°049′ N, 125°429′ E), 271 F_2_ progenies, M4-5 (n = 10), M1-15 (n = 10), and their F_1_ (n = 10) hybrids were planted in 2021. In 2022, F_2_ (n = 393) and M4-5 (n = 10), and M1-15 (n = 10) and F_1_ (n = 10) were planted in the greenhouse of the Facility Horticulture Engineering Center, Northeast Agricultural University, Harbin (45°774′ N, 126°727′ E), China. For Harbin’s normal climatic circumstances, watering, extirpating weed, and vermin control were performed in accordance with industry standards. According to the climacteric or non-climacteric behavior of the fruit, different combinations of harvesting indicators are used for fruit harvesting. In the fruits of our F_2_ population, there are both climactic and non-climactic fruits. Our harvesting index is based on the method of Obando-Ulloa et al. [30], wherein the melon fruit is picked when the fruit pedicel begin to split and an aromatic scent can be smelled.

### 2.2. Determination of Fruit Traits

A GY-4 digital fruit firmness tester with an 11 mm diameter probe (Aipli, China) was used to determine the firmness of the inner and outer peels of the same fruit three times each, and the average value calculated was represented as the hardness index (kg/cm^2^). The juice in the middle of the fruit was taken and measured with a BM-02 digital display refraction instrument (Dongmei, China); light was avoided during the measurement. All measurements were repeated three times, and the average value was recorded.

### 2.3. Genomic DNA Sequencing

Fresh, 2-week-old leaves of the M4-5, M1-15, F_1_ and F_2_ populations were quick-frozen in liquid nitrogen, and gDNA was extracted using a modified cetyltrimethylammonium bromide (CTAB) method [31]. DNA concentration and quality were then determined using SMA3000 spectrophotometer (Plextech, Shenzhen, China) and 1% agarose gel electrophoresis, respectively. On a high-throughput Illumina sequencing platform, libraries prepared from the genomic DNA of the two parents, M4-5 and M1-15, was sequenced. 

### 2.4. SNP Development, Annotation and CAPS Marker Development

The high-quality resequencing data of the parental were obtained using the preprocessing software FASTX-Toolkit (v0.0.13). The resequencing data were aligned with the Melon (DHL92) v3.6.1 Genome [32] using the Burrows–Wheeler Aligner (BWA). The parental resequencing data were aligned with the melon reference genome to the identified SNP sites using SAMtools (v1.12)-mpileup software. SNPs in the resequencing data were detected using VarScan (v2.0) to generate VCF files. SnpEff (v4.3) was used to export SNPs to web-based generated HTML, and according to their genetic variation effects introns, exons, start-stop codons, upstream-downstream regions, splice regions, and 5’ to 3’ end UTR regions for annotation.

For the development of CAPS markers, a total of 10–15 random SNP sites before and after 500 base pair sequences with suitable restriction endonucleases were mined across each chromosome using the SNP2CAPS [33]. The potential SNP sequences were transformed into CAPS markers following the manual settings of molecular parameters of the Primer Premier (v6.0) program.

### 2.5. CAPS Genotype Analysis

The CAPS primers were designed using Primer Premier (v6.0) program (The CAPS marker primer sequences used to construct the genetic linkage map are listed in Supplementary Table S1) and PCR amplification was performed for the parents and F_1_ population. The obtained PCR products were cut to detect polymorphisms in the parents and F_1_ population. CAPS markers with uniform location distribution and clear bands were selected for genotyping of the F_2_ population. The components and concentrations of the PCR reaction system are shown in Supplementary Table S2. Three restriction enzymes were used to cleave the PCR products (*Eco*R I, *Hin*d III, and *Bam*H I, 10 U/μL, TAKARA). According to the manufacturer’s instructions, a mixture of 5 μL of PCR product, 0.2 μL of restriction enzyme (10 U/μL), 1.5 μL of enzyme-specific buffer, and 8.3 μL of sterile double distilled water was used for reaction in a 37 °C incubator for 4–5 h. All CAPS markers and products of both parents and F_1_ generation were verified and examined using electrophoresis with 1% agarose gel. Images from gel electrophoresis were obtained using an image analysis system (Champ Gel 6000, Saizhi Entrepreneurship, Beijing, China).

### 2.6. Genetic Linkage Map Construction

QTL IciMapping (v4.0) and R/qtl (v1.5) [34] were used to construct linkage maps for the F_2_ population. The genome-wide LOD threshold at α = 0.05 was estimated using 1000 repeat replacement tests. LOD > 2.5 is used as the threshold for detecting the presence of QTLs. Composite interval mapping was used to scan the whole genome at a walking speed of 1.0 cM. The identified QTLs were coded using abbreviations for the traits, followed by the linkage group number and QTL number.

### 2.7. Statistical Analysis

All data were presented as mean ± SD and ranges. Analyses of distributions and correlations were performed using R (v4.2.0), for the correlation analysis of SSC, EF, and EPF. SPSS (v23.0) was used for the analysis of fruit quality traits in the parental lines and the F_1_ and F_2_ generations. Frequency distributions of SSC, EF, and EPF in the M4-5 and M1-15 derived F_2_ populations were calculated based on counts. Microsoft Excel (v2021) was used to record band information and trait data (SSC, EF, and EPF) after enzyme digestion. GraphPad Prism (v8.0) software was used for *SSC6.1* CAPS marker genotype and SSC phenotype analysis.

## 3. Results

### 3.1. Phenotyping of Melon Morphological Traits

**Figure 1:**
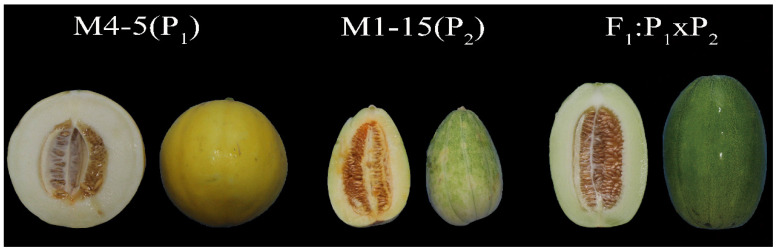
A picture of the melon parents (P_1_ M4-5, P_2_ M1-15) and F_1_ progeny, showcasing their corresponding fruit longitudinal and lateral profiles.

Two melon parental lines, M4-5 (P_1_, female) and M1-15 (P_2_, male), were selected as the experimental materials. The exocarp and pericarp firmness of M4-5 was 14.51 ± 0.37 kg/cm^2^ and 4.17 ± 0.18 kg/cm^2^, respectively; the SSC for M4-5 was 8.64 ± 0.30%. The exocarp and pericarp firmness of M1-15 was 14.11 ± 0.13 kg/cm^2^ and 6.11 ± 0.14 kg/cm^2^, respectively; the SSC for M1-15 was 10.21 ± 0.30%. The outer and longitudinal sections of both parents and F_1_ melons are shown in Figure 1.

#### 3.1.1. Exocarp Firmness (EF) and Edible Pericarp Firmness (EPF)

**Figure 2:**
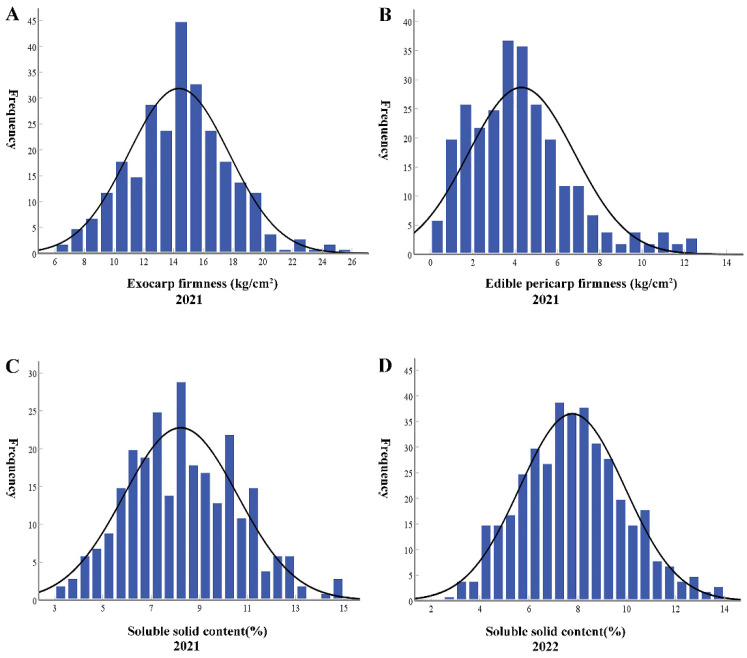
Distribution of EF (**A**), EPF (**B**), SSC 2021 (**C**), and SSC 2022 (**D**) frequencies in the F_2_ populations.

EF of the two parental lines were quite similar, 14.51 ± 0.37 kg/cm^2^ and 14.11 ± 0.13 kg/cm^2^ for M4-5 and M1-15, respectively. The EF of the F_1_ generation (15.46 ± 0.17 kg/cm^2^) was higher than that of M4-5 and M1-15 (Table 1). The EF of the F_2_ population was diverse, ranging from 6.20 kg/cm^2^ to 25.20 kg/cm^2^ (Figure 2A). M4-5 and M1-15 had an EPF of 4.17 ± 0.18 kg/cm^2^ and 6.11 ± 0.14 kg/cm^2^, respectively. The F_1_ generation had an EPF of 5.14 ± 0.10 kg/cm^2^, which was comparable to the parental lines (i.e., M4-5 and M1-15). The EPF of the F_2_ population was diverse, ranging from 2.60 kg/cm^2^ to 14.60 kg/cm^2^ (Figure 2B).

#### 3.1.2. Soluble Solid Content (SSC)

In a previous study [29], SSC of the parental lines, i.e., M4-5 and M1-15 lines, and the F_1_ population were 9.24 ± 0.43%, 10.69 ± 0.26%, and 7.74 ± 0.58%, respectively (Table 2). In the F_2_ population, SSC ranged from 4.00% to 14.00% (Supplementary Figure S1).

In 2021, we measured the SSC of the parents and their F_1_ and F_2_ populations. SSC of M1-15 (10.21 ± 0.30%) was higher than that of M4-5 (8.64 ± 0.30%). SSC of F_1_ (7.93 ± 0.27%) was lower than that of M4-5 and M1-15 (Table 2). SSC of the F_2_ population was diverse, ranging from 2.60% to 14.60% (Figure 2C).

In 2022, we further measured the SSC of the parents and their F_1_ and F_2_ populations. The SSC value of the two parents and F_1_ population in 2022 was consistent with that from the previous two years (i.e., 2018 and 2021). However, the SSC value of the F_2_ population in 2022 was lower than that of the previous two years. SSC of the F_2_ generation showed continuous normal distribution (Figure 2D, Table 2).

#### 3.1.3. Correlation Analysis

The correlation analysis showed that these fruit traits have a good relationship with each other (Table 3). Specifically, SSC was significantly correlated with EF, whereas EPF and EF, and SSC and EPF, were negatively correlated with each other.

### 3.2. Single Nucleotide Polymorphisms (SNPs) and CAPS Marker Analysis

**Figure 3:**
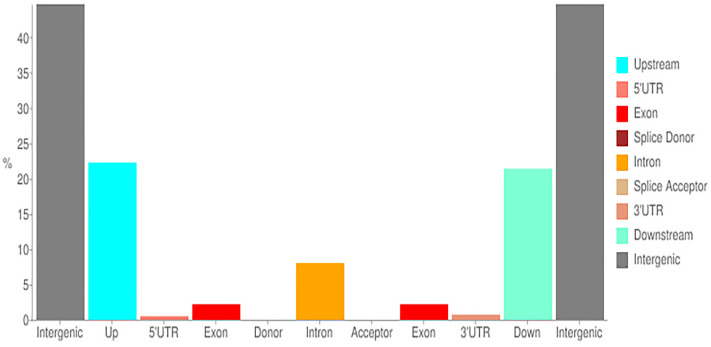
Location of various SNP variations found in newly sequenced areas of parental lines of melon. The *x*-axis represents the regions of distribution, and *y*-axis represents the proportion of SNPS in each location.

The genome sequencing revealed 53.85% non-synonymous SNPs and 46.15% polymorphic SNPs, of which 44.68%, 21.39%, 22.27%, 0.01%, 0.01%, 0.17%, 0.74%, 0.51%, 8.01%, and 2.21% were located in the intergenic, downstream, upstream, splice acceptor, splice donor, splice region, 3′ UTR, 5′ UTR, intron, and exon regions, respectively (Figure 3).

**Figure 4:**
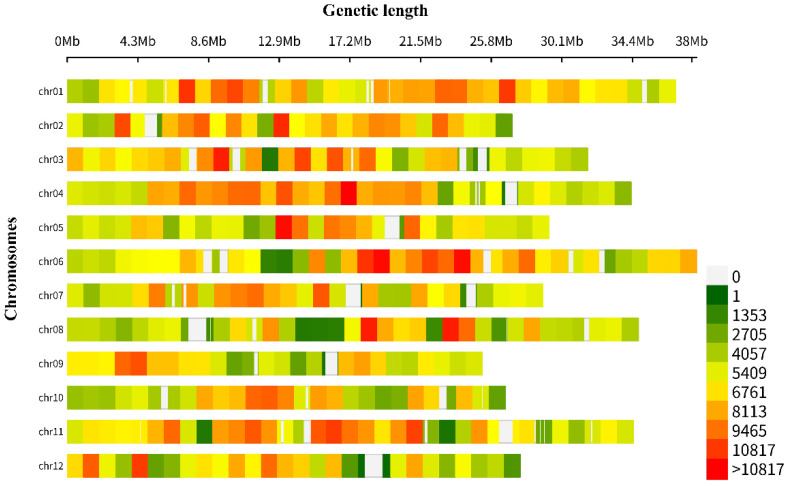
Total SNP variants detected within a 1-Mb window-sized chromosomal region of resequenced parental lines.

The total length of the whole genome was 375,360,399 bp. The SNP variant density was calculated in the chromosome region with a 1-Mb size window. A total of 2,388,036 SNP variants were detected, of which the highest number of SNP mutations were found on chromosome 1 (270,720), and the lowest number of SNP mutations on chromosome 9 (150,871). The SNP variant density in the whole genome was calculated using the CMplot R package, and the sequences of the two parental lines were compared with that of the melon reference genome (v3.6.1) (Supplementary Table S3, Figure 4).

A total of 290 CAPS markers were designed, verified by P_1_, P_2_ and F_1_, of which 116 CAPS markers were polymorphic, and the polymorphic rate was 40%. The restriction endonucleases (*Eco*R I, *Hin*d III, and *Bam*H I, 10 U/μL, TAKARA) were used to cut the PCR products, which were examined using electrophoresis with 1% agarose gel.

### 3.3. Construction of Genetic Linkage Map

**Figure 5:**
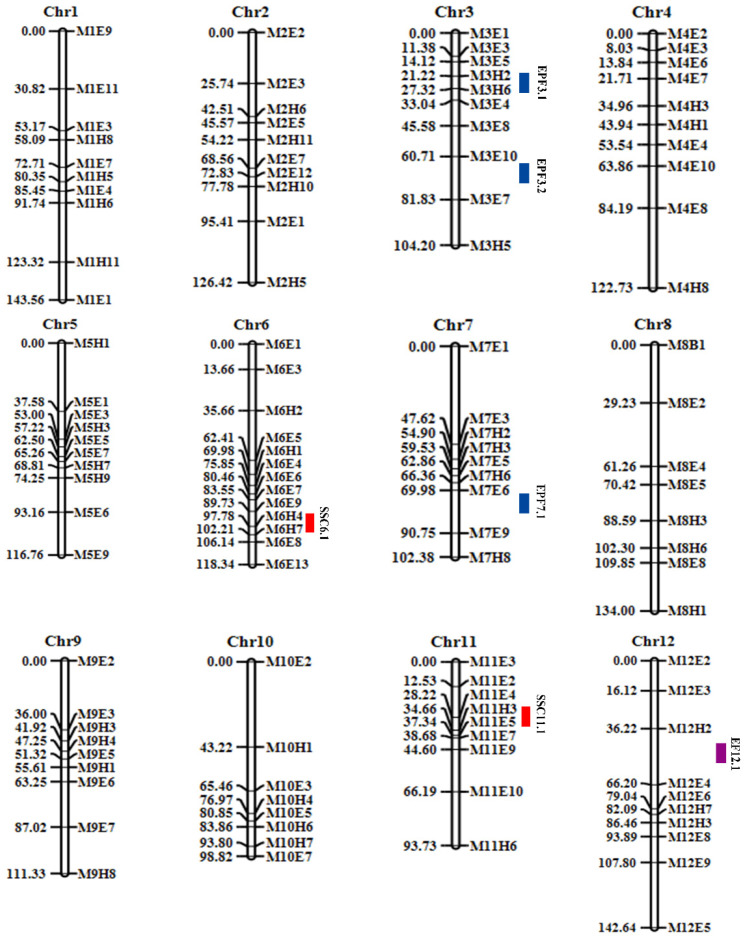
Based on the genotyping of 116 SNP-CAPS markers in the F_2_ population, the genetic map was established. Purple (EF), blue (EPF), and red (SSC) represent mapped QTL areas. The genetic location of each chromosome is aligned on the left side, and the marker position on each chromosome is aligned on the right side.

The genetic linkage maps of the 2021 F_2_ populations of M4-5 and M1-15 were constructed with the 116 SNP-CAPS markers (Figure 5). Table 3 shows the marker distribution on each chromosome. The linkage map consisted of 12 linkage groups, with the largest number of CAPS markers located on chromosome 6, which has a total length of 118.34 cM and an average CAPS marker distance of 9.10 cM. The average genetic distance of the 116 CAPS markers was 12.20 cM, whereas the overall genetic distance of the 12 linkage maps (whole genome) was 1414.88 cM (Table 4).

### 3.4. QTLs Related to the Quality Traits of Melon

Using the melon genotype and phenotype data, six putative QTLs potentially related to melon fruit traits were identified, which are distributed on multiple chromosomes (Table 5; Figure 5). Specifically, two QTLs (*SSC6.1* and *SSC11.1*) were mapped on chromosomes 6 and 11 (Table 5 and Figure 5). Among all the six QTLs, *SSC6.1* had the highest logarithm of odds (LOD) score of 9.81, with an additive effect of −1.3466 and percentage phenotype variance (PVE%) of 16.32. It was positioned at 18 cM from the starting site of the flanking CAPS markers, *M6H4-M6E8* (21,063,516 bp–31,537,693 bp) (genetic confidence interval of 8.36 cM and physical interval of 10.47 Mb).

**Figure 6:**
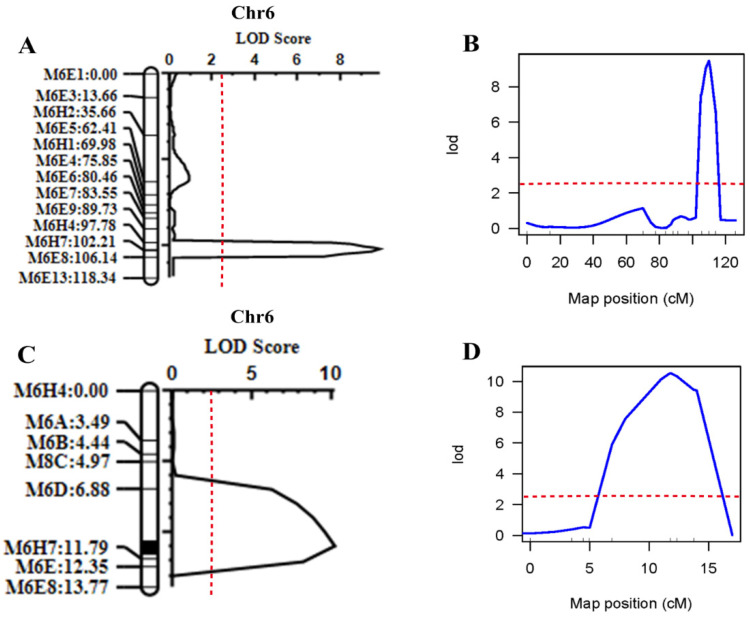
Positioning intervals for 2021 and 2022 SSC. (**A**) QTL IciMapping software 2021 QTL mapping results. (**B**) R/QTL software 2021 QTL mapping results. (**C**) QTL IciMapping software 2022 QTL mapping results. (**D**) R/QTL software 2022 QTL mapping results. The red dotted line indicates an LOD value of 2.5.

Two different QTL software (QTL IciMapping (v4.0) and R/qtl v1.5), identified a major effective QTL between CAPS markers *M6H4* and *M6E8* that is closely linked to the SSC trait. The intervals of CAPS markers in both software were roughly the same (Figure 6A,B).

To locate the *SSC6.1* more precisely, based on QTL analysis in 2021, a larger F_2_ population (n = 393) was planted in 2022 and eight new CAPS markers were identified in the flanking regions of the *M6H4-M6E8* markers. As a result, the range was narrowed to *M6D-M6E* (25,232,480–28,148,360 bp), and the physical distance was narrowed to 2.92 Mb (Figure 6C). The intervals (i.e., *M6H4-M6E8*) obtained using both software, i.e., R/qtl and QTL IciMapping, were the same (Figure 6D). The consistent interval calculated using the two software was similar, with LOD scores of 10.19 and 10.5, respectively.

On chromosome 12, a QTL *(EF12.1*) linked to EF was found at 56 cM (Table 5; Figure 5). The genetic distance between markers *M12H2* and *M12E4* was 29.98 cM, and a total of 6.45% PVE was detected at an LOD score of 2.77.

Three QTLs (*EPF3.1*, *EPF3.2*, and *EPF7.1*) were identified on chromosomes 3 and 7 (Table 5; Figure 5), with LOD scores of 3.56, 3.58, and 2.90; the additive effect values of 0.0041, 0.7995, and 0.9399; and the PVE% values of individual effect of 6.14, 5.64, and 4.89, respectively. On chromosome 3, the two QTLs for *EPF3.1* and *EPF3.2* were identified, with locations at 26 cM and 61 cM, and the genetic distance between the two markers at 11.82 cM (*M3H2-M3E4*) and 36.25 cM (*M3E8-M3E7*), respectively. One QTL (*EPF7.1*) is located at 75 cM on chromosome 7, and the genetic interval of the flanking markers *M7H6-M7E9* is 24.39 cM.

### 3.5. Marker Verification

**Figure 7:**
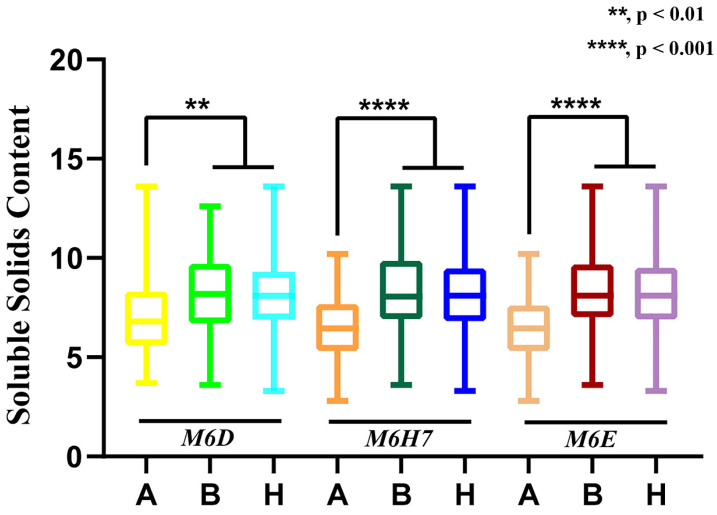
Three markers were used to conduct a correlation analysis between phenotypes and genotypes. The *X*-axis represents the three genotypes (A, B and H) of CAPS markers, and the *Y*-axis represents the value of SSC. Asterisk symbols (**, ****) represents the statistical significance; **, *p* < 0.01, ****, *p* < 0.001.

Three markers flanking mapped QTL region and linked to the fruit SSC (Figure 7) were used to analyze the genotype–phenotype correlation of 2022 F_2_ population to verify the accuracy of the QTL mapping results. The P_1_, P_2_, and F_1_ populations had genotype A (low SSC), genotype B (high SSC), and genotype H (SSC between P_1_ and P_2_), respectively. In the F_2_ population, the significance of *M6H7* and *M6E* among the three markers was most prominent, showing a greater probability of QTL between these two loci. These results further demonstrated the reliability of the QTL findings.

## 4. Discussion

Melon, a popular, horticultural crop, exhibits significant phenotypic variations. DNA-based genetic markers in the last few decades has been used to elucidate fruit quality traits in watermelon, melon and cucumber [35,36,37,38,39]. In this present study, we constructed a genetic linkage map using F_2_ mapping populations over a three-year period at three distinct locations using genome-wide SNP-CAPS markers, and genetically mapped putative loci contributing to melon traits, such as exocarp and pericarp firmness and SSC (Figure 5).

In addition to the influence of external factors, such as cultivation environment and agronomic measures, the complex quantitative feature of sugar buildup in melon fruit is reportedly regulated by several genes [40]. In one study aimed to identify the genetic loci responsible for the sugar content, many QTLs and genes related to sugar content in melon fruit were identified [41]. However, at present, the reported QTLs related to the sugar content trait in melon can only explain a low level of phenotypic variation, which is unstable in different generations and under different environments and can interact with environmental factors, severely limiting the in-depth study of genetic mechanisms regulating this trait [42,43]. In previous studies, QTLs for SSC were found on chromosomes 2, 6, 7, and 9 [29]. However, in our 2021 and 2022 experiments, QTLs for SSC were found only on chromosome 6, in contrast to previous trials [29], and no QTLs for SSC were found on chromosomes 2, 7, and 9. Thus, we hypothesize that environmental factors may affect the phenotypes and interfere with the identification of the genetic loci contributing to the SSC trait.

Fruit SSC serves as a useful indicator for melon maturity. Chace et al. [44] proposed SSC as an objective quality indicator, and Mutton et al. [45] showed that both SSC and pulp firmness are key indicators for evaluating the quality of melons. Soluble solid content is usually expressed in % [45]. According to UNECE [46], SSC in the middle of the flesh of Charentais melons is ≥10%, whereas that of other types is ≥8%. In this experiment, the SSC ranges of the P_1_, P_2_, F_1_, and F_2_ populations were 8.64–9.73%, 10.21–10.85%, 7.74–7.93%, and 2.8–14.6%, respectively. According to several cultivar experiments, the reported SSC range of Galia melons was 10.2–16.1%, of melons was 11.0–14.6%, of Oriental crisp melons was 9.7–17.2%, of Canary melons was 10.4–15.2%, and of oriental melons was 10.5–14.2% [47,48,49].

The type, content, and composition of sugar can significantly affect fruit quality [5,50]. Thus, it is of practical significance to understand the genetic mechanism regulating sugar content in melon, which may be used in the breeding of high-quality melon varieties. Thus far, researchers have identified many QTLs in the sugar accumulation trait in melons, suggesting that this trait is highly complex and can be regulated by multiple genes [51,52]. Using the F_2_ and DHL populations from a hybrid between the Piel de Sapo (PS) variety and the Korean germplasm PI161375 and using composite interval mapping, five QTLs related to SSC were identified [43]. By generating RILs from the parents of the melon varieties, TopMark and USDA-846-1, ten SSC-related QTLs were identified [22]. In the present study, two SSC related QTLs were identified and located on chromosomes 6 and 11 from the experiment performed in the first year (2021), with LOD and PVE% of 9.81 and 16.32% for *SSC6.1*, respectively. From the experiment performed in the second year, we have developed eight CAPS markers around SSC6.1, and expanded the F_2_ population, which yielded significant results, with LOD and PVE% of 10.19 and 12.30%, respectively, exerting a strong potential influence on SSC.

Several mapping populations were established to investigate SSC traits in melon, and many QTLs related to the fruit sugar content trait were discovered. Currently, reported QTLs related to melon sugar content traits can only explain the low levels of phenotypic variation and exhibit inconsistency across different generations and environments; strong genotype–environment interaction further makes it challenging to investigate the genetic mechanism regulating this trait [42]. In this study, using SNP–CAPS markers established from the F_2_ populations, and a QTL mapping strategy at three sites over a period of three years, we identified a stable QTL (*SSC6.1*) for SSC (The research results of 2018 are shown in Supplementary Figure and Table).

The exocarp and pericarp firmness of melons is particularly important for consumer choice as it is closely related to transportation and shelving. In particular, EF, which relates to shelf life and market price, is a significant index. Fruit firmness is an illustration of a complex quantitative attribute impacted by genes that regulate cell wall, cell dilatation, and stratum trait [53,54,55]. In a study by Moreno et al. [11], five QTLs (*FF2.2*, *FF3.5*, *FF8.2*, *FF8.4*, and *FF10.2*) related to flesh firmness were detected using PI 161375 (SC) and Piel de Sapo (PS). In another study by Harel-Beja et al. [50], using the RIL population developed by crossing two melon subspecies, PI414723 (*agrestis* subspecies) and Dulce (*melo* subspecies), they identified QTLs related to the fruit firmness on chromosomes 1 and 5. Herein, three QTLs related to fruit firmness were identified on chromosomes 3 and 7, including two QTLs present on chromosome 3. The LOD score of the two QTLs on chromosome 3 was 3.58 and 3.56, respectively, higher than that of the one on chromosome 7 (2.90). Among these, the position of *EPF3.2* is similar to that of *FF3.5* in the previous study by Moreno et al. [11].

Numerous studies have thoroughly examined the influences of genetic factors on traits, such as melon fruit firmness and other qualities. According to Beaulieu and Lea [56], melons get softer by 51.9% in 13 days between blooming and harvesting, with nearly a third of the softening process occurring between 35 and 38 days after flowering. The ethylene (ETH)-dependent regulation of cell wall-modifying proteins involved in melon ripening is linked to the maturation of ETH production. This is demonstrated by the observation that inhibition of 1-aminocyclopropane-1-carboxylate oxidase (ACO) gene expression can inhibit the hardness of transgenic fruits. However, after exogenous ETH administration, ACO expression was recovered and phenotype was restored [57]. Moreover, treatment with the ETH inhibitor 1-methylcyclopropene can delay the softening process of physiologically ripe melon fruit [58]. In previous studies [11], *FF3.5* and ETH (*ETH3.5*) were both identified on chromosome 3; *FF3.5* was a QTL related to fruit firmness, and *ETH3.5* was a QTL related to ETH content of fruits at maturity, leading to softening and reduction in fruit firmness. The QTL (*EPF3.2*) identified in our study was similar to *FF3.5* [11] (i.e., two QTLs positions are similar), indicating the reliability of our results.

Recent studies on watermelons have shown that tissue lignification promotes the formation of pericarp stone cells, thus increasing the firmness of the pericarp [59]. Different studies have been conducted exploring the molecular mechanisms underlying fruit firmness, mainly focusing on the ripening of fruit and the softening of cell walls caused by increased enzyme activity [53,60]. For example, β-d-xylosidase is involved in the breakdown of xylans and regulates fruit development and ripening in tomatoes [61] and Japanese pears [62]. This gene might also contribute to melon fruit firmness by controlling skin thickness and external pressure. Potential genes that can provide further molecular insights for upcoming CAPS marker-assisted selection breeding may be identified among the genetic loci we have identified.

## 5. Conclusions

NGS data can significantly improve the validity of genetic linkage maps and CAPS markers. Our constructed genetic linkage map covered 12 chromosomes, with a total genetic distance of 1414.88 cM. We mapped six QTLs related to fruit quality traits (EF, EPF and SSC). The presence of the QTL, *SSC6.1*, was verified in the F_2_ populations in three independent experiments and at three different locations, which can facilitate MAS of potential genes in the breeding of different melon varieties.

## Figures and Tables

**Table 1:** Traits data analysis of EF (kg/cm^2^) and EPF (kg/cm^2^) in P_1_, P_2_, F_1_ and F_2_ generations (2018 [29], 2021, 2022).

Traits	Years	P_1_	P_2_	F_1_	F_2_			
					Mean ± SD	Range	Kurtosis	Skewness
EF	2018	14.29 ± 0.34	14.12 ± 027	15.92 ± 0.76	14.64 ± 2.18	8.50–26.00	4.23	1.06
	2021	14.51 ± 0.37	14.11 ± 0.13	15.46 ± 0.17	14.35 ± 2.10	6.20–25.50	0.43	0.28
	2022	14.40 ± 0.27	13.56 ± 0.20	15.72 ± 0.45	17.87 ± 0.24	8.30–27.50	−0.11	−0.12
EPF	2018	4.44 ± 0.37	6.06 ± 0.17	4.93 ± 0.23	5.15 ± 1.64	1.00–10.60	0.27	0.63
	2021	4.17 ± 0.18	6.11 ± 0.14	5.14 ± 0.10	8.23 ± 2.37	2.60–14.60	−0.25	0.17
	2022	4.37 ± 0.21	6.28 ± 0.20	5.03 ± 0.33	4.25 ± 0.09	1.20–8.40	0.32	−0.98

**Table 2:** Mean ± standard deviation (SD) and range of SSC (%) in the P_1_, P_2_, F_1_, and F_2_ generations over three years.

Year	P_1_	P_2_	F_1_	F_2_			
				Mean ± SD	Range	Kurtosis	Skewness
2018 [29]	9.24 ± 0.43	10.69 ± 0.26	7.74 ± 0.58	8.30 ± 2.20	4.00–14.00	−0.28	0.44
2021	8.64 ± 0.30	10.21 ± 0.30	7.93 ± 0.27	8.23 ± 2.37	2.60–14.60	−0.25	0.17
2022	9.73 ± 0.17	10.85 ± 0.07	7.77 ± 0.11	7.77 ± 2.15	2.80–13.60	−0.21	0.23

**Table 3:** Correlation analysis of fruit traits in the F_2_ plant population. *, *p* < 0.05.

Traits	SSC	EPF	EF
SSC	1	–	–
EPF	−0.02	1	–
EF	0.12 *	−0.06	1

**Table 4:** Details of the genetic linkage map (2021).

Chr No.	Marker No.	Linkage Group Distance (cM)	
		Genetic Distance	Average Distance
01	10	143.56	14.35
02	10	126.42	12.64
03	10	104.20	10.42
04	10	122.73	12.27
05	10	116.76	11.68
06	13	118.34	11.83
07	9	102.38	11.38
08	8	134.00	16.75
09	9	111.30	12.37
10	8	98.82	12.35
11	9	93.73	10.41
12	10	142.64	14.26
Total	116	1414.88	12.20

**Table 5:** QTLs information of EF, EPF, and SSC.

Traits	QTLs	Chr No.	Position (cM)	Marker Interval	Interval Mapping		Phenotypic Effect	
					LOD	PVE%	Additive	Dominance
EF	*EF12.1*	12	56	*M12H2-M12E4*	2.77	6.45	0.6332	1.8113
EPF	*EPF3.1*	3	26	*M3H2-M3E4*	3.56	6.14	0.0041	1.24
	*EPF3.2*	3	61	*M3E10-M3E7*	3.58	5.64	0.7995	0.42
	*EPF7.1*	7	75	*M7H6-M7E9*	2.90	4.89	0.9399	−0.3579
SSC	*SSC6.1*	6	102	*M6H4-M6E8*	9.81	16.32	−1.3466	−0.0064
	*SSC11.1*	11	37	*M11H3-M11E7*	2.72	6.27	−0.7174	0.0751

## Data Availability

Data will be made available upon request from the corresponding author.

## Supplementary Materials

The following supporting information can be downloaded at: https://www.mdpi.com/article/10.3390/cimb45040224/s1, Supplementary Figure and Table: The position of the fruit traits QTL in the genetic linkage map in the previous research and QTL information for fruit traits. Supplementary Figure S1: Frequency distribution of BRC in F2 population derived from M4-5 and M1-15 (2018). Supplementary Table S1: CAPS makers primer sequence. Supplementary Table S2: The PCR reaction system. Supplementary Table S3: Summary of detected SNP variants distribution in re-sequenced parental lines of melon.
