# Supplementary material for: Genetic Mapping and QTL Analysis of Fruit Traits in Melon (Cucumis melo L.)
Source: Curr Issues Mol Biol. 2023 Apr 14;45(4):3419–33. doi: 10.3390/cimb45040224 (PMC10137213; doi:10.3390/cimb45040224)
Supplement: Supplementary file 1 [file cimb-45-00224-s001.zip › Supplementary Figure and Table.pdf]

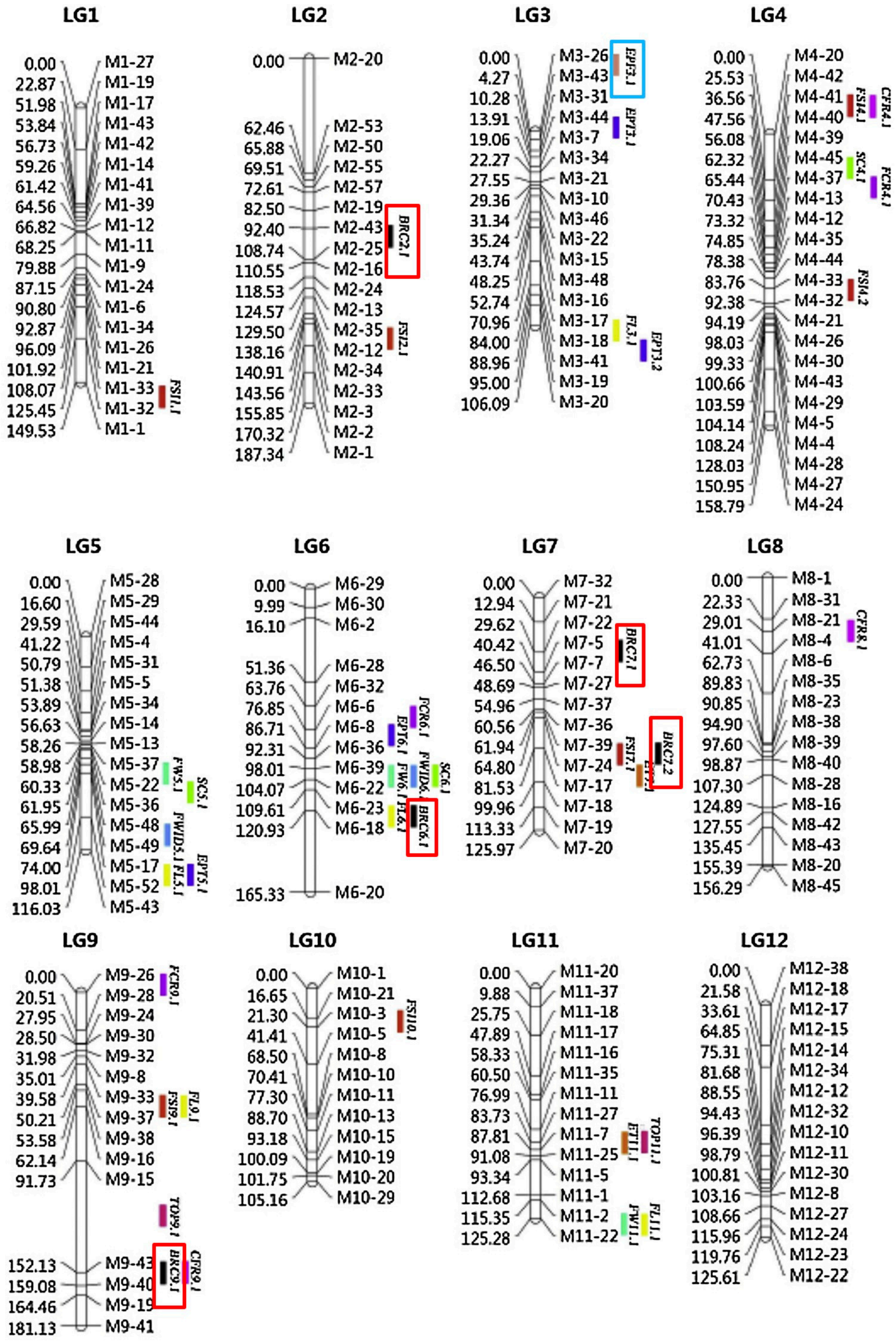

Supplementary Figure. The position of the fruit traits QTL in the genetic linkage map in the previous research[1], the red box indicates the QTL of soluble solid content, and the blue box indicates the QTL position of edible Pericarp firmness.

Supplementary table. QTL information for fruit traits in 2018.

Trait	QTL	LG	Adjacent markers	Position	Marker rang	LOD	Additive	(%)
Brix content	BRC2.1	2	M2–43 / M2–25	108	108.74–110.55	2.8176	0.645	4.1983
	BRC6.1	6	M6–23 / M6–18	111	109.61-120.93	2.8073	0.6274	4.3069
	BRC7.1	7	M7–5 / M7–7	45	40.42-46.50	2.6752	0.5011	4.02
	BRC7.2	7	M7–39 / M7–24	62	61.94-64.80	2.8363	0.4484	3.8933
	BRC9.1	9	M9–43 / M9–40	159	159.08-164.46	3.991	−0.7071	5.7898
Edible Pericarp firmness	EPF3.1	3	M3–26 / M3–43	4	0-4.27	2.6119	−0.4767	4.1761

1. Zhang, T.; Ding, Z.; Liu, J.; Qiu, B.; Gao, P. QTL mapping of pericarp and fruit-related traits in melon (Cucumis melo L.) using SNP-derived CAPS markers. *Scientia Horticulturae* **2020**, *265*, doi:10.1016/j.scienta.2020.109243.
