# Supplementary material for: Genetic Mapping and QTL Analysis of Fruit Traits in Melon (Cucumis melo L.)
Source: Curr Issues Mol Biol. 2023 Apr 14;45(4):3419–33. doi: 10.3390/cimb45040224 (PMC10137213; doi:10.3390/cimb45040224)
Supplement: Supplementary file 1 [file cimb-45-00224-s001.zip › Supplementary Figure S1.pdf]

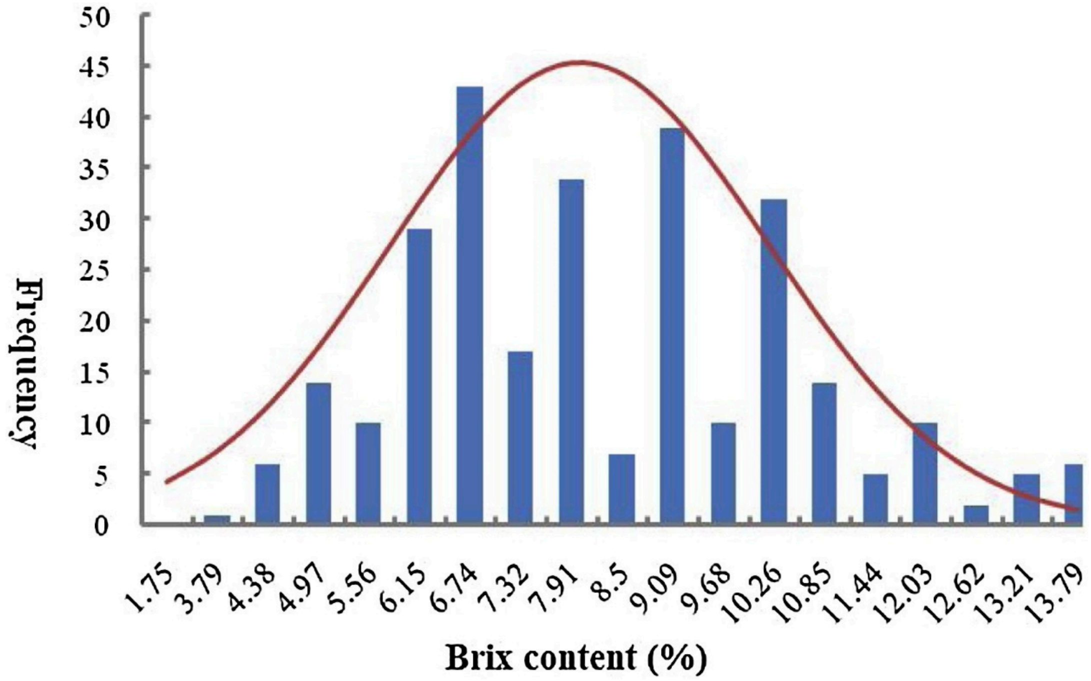

Supplementary Figure S1. Frequency distribution of BRC in F_2 population derived from M4–5 and M1–15 (2018).
